# Characterization of an N-Terminal Non-Core Domain of RAG1 Gene Disrupted Syrian Hamster Model Generated by CRISPR Cas9

**DOI:** 10.3390/v10050243

**Published:** 2018-05-06

**Authors:** Jinxin Miao, Baoling Ying, Rong Li, Ann E. Tollefson, Jacqueline F. Spencer, William S. M. Wold, Seok-Hwan Song, Il-Keun Kong, Karoly Toth, Yaohe Wang, Zhongde Wang

**Affiliations:** 1 Department of Pathology, School of Basic Medical Sciences, Academy of Medical Sciences, Zhengzhou University, Zhengzhou 450052, China; jinxin.miao@yahoo.com; 2 Department of Animal, Dairy, and Veterinary Sciences, Utah State University, Logan, UT 84322, USA; lirong14@gmail.com; 3 Department of Molecular Microbiology and Immunology, Saint Louis University School of Medicine, St. Louis, MO 63104, USA; baoling.ying@health.slu.edu (B.Y.); ann.tollefson@health.slu.edu (A.E.T.); jacqueline.spencer@health.slu.edu (J.F.S.); bill.wold@health.slu.edu (W.S.M.W.); 4 Department of Animal Science, Division of Applied Life Science (BK21Plus), Graduate School of Gyeongsang National University, Jinju 52828, Korea; siwd2002@gmail.com (S.-H.S.); ikong7900@gmail.com (I.-K.K.); 5 Institute of Agriculture and Life Science, Gyeongsang National University, Jinju 52828, Korea; 6 Centre for Molecular Oncology, Barts Cancer Institute, Queen Mary University of London, London EC1M 6BQ, UK; 7 Auratus Bio, LLC., Canton, SD 57104, USA

**Keywords:** Syrian hamster, RAG1, SCID, CRISPR/Cas9, animal model, adenovirus infection

## Abstract

The accumulating evidence demonstrates that Syrian hamsters have advantages as models for various diseases. To develop a Syrian hamster (*Mesocricetus auratus*) model of human immunodeficiency caused by *RAG1* gene mutations, we employed the CRISPR/Cas9 system and introduced an 86-nucleotide frameshift deletion in the hamster *RAG1* gene encoding part of the N-terminal non-core domain of RAG1. Histological and immunohistochemical analyses demonstrated that these hamsters (referred herein as *RAG1-86nt* hamsters) had atrophic spleen and thymus, and developed significantly less white pulp and were almost completely devoid of splenic lymphoid follicles. The *RAG1-nt86* hamsters had barely detectable CD3^+^ and CD4^+^ T cells. The expression of B and T lymphocyte-specific genes (CD3γ and CD4 for T cell-specific) and (CD22 and FCMR for B cell-specific) was dramatically reduced, whereas the expression of macrophage-specific (CD68) and natural killer (NK) cell-specific (CD94 and KLRG1) marker genes was increased in the spleen of *RAG1-nt86* hamsters compared to wildtype hamsters. Interestingly, despite the impaired development of B and T lymphocytes, the *RAG1-86nt* hamsters still developed neutralizing antibodies against human adenovirus type C6 (HAdV-C6) upon intranasal infection and were capable of clearing the infectious viruses, albeit with slower kinetics. Therefore, the *RAG1-86nt* hamster reported herein (similar to the hypomorphic *RAG1* mutations in humans that cause Omenn syndrome), may provide a useful model for studying the pathogenesis of the specific RAG1-mutation-induced human immunodeficiency, the host immune response to adenovirus infection and other pathogens as well as for evaluation of cell and gene therapies for treatment of this subset of RAG1 mutation patients.

## 1. Introduction

Recombination activating gene 1 (*RAG1*), as well as *RAG2*, is required for the activation of immunoglobulin (Ig) and T cell receptor (TCR) genes by catalyzing the combinatorial joining of variable (V), diversity (D), and joining (J) gene segments that encode the antigen-recognition sequences of the Ig genes in B cells and TCR genes in the T cells, respectively [1]. The RAG1 and RAG2 proteins form a heterotetramer that recognizes the Recombination Signal Sequence (RSS) flanking the V, D, and J regions of Ig genes or TCR genes and nicks the DNA to induce the DNA recombination events [2]. Mutations in the *RAG1* or *RAG2* genes may lead to a failure of V(D)J recombination of Ig and TCR genes and are the prominent causes for severe combined immunodeficiency (SCID), such as Omenn syndrome, a form of primary immunodeficiency characterized by abnormal development of functional T cells and B cells [3]. The adaptive immune system in SCID patients is defective both in antibody response by B cells and a lack of functional T cells. Consequently, these patients are highly prone to severe bacterial, viral, or fungal infections early in life and may fail to thrive. Depending on the types of genetic mutations in the *RAG1* or *RAG2* genes, autoimmunity associated with expansion of oligoclonal T cells and production of autoantibodies is also often observed in affected patients [4].

Animal models in which the *RAG1* or *RAG2* gene is genetically inactivated have contributed greatly to the understanding of SCID caused by the loss of function mutations in the *RAG* genes. The most often used animal models are the *Rag1* and *Rag2* knockout (KO) mice and rats, which are characterized by the absence or abnormality of B and T lymphocyte development, autoimmunity, and inability to mount adequate adaptive immunity to infections [5]. Given that mice or rats are not suitable hosts of certain viruses [6,7], other animal models are needed. Recently, to overcome the sole reliance on the murine models, *RAG* KO animal models from other species, such as rabbits [8] and pigs [9,10], have also been produced. While these large animal models may provide new insights into the pathogenesis of diseases caused by loss of function of the RAG genes, a conceivable drawback of these animal models is the high cost associated with them. More importantly, because not all SCID patients experience a total loss of function of the *RAG1* or *RAG2* gene, gene KO animal models alone cannot recapitulate the entire spectrum of genetics and pathologies of these patients. For example, about 19% of the genetic mutations in the *RAG1* gene that cause Omenn syndrome occur in the sequences of the *RAG1* gene encoding the non-core domain of RAG1 [11]. Some of the genetic mutations in both *RAG1* or *RAG2* genes are hypomorphic which, while significantly reducing the V(D)J recombination activities of the RAG proteins, do not result in a complete failure of V(D)J recombination. Therefore, animal models carrying the identical or similar genetic mutations in the *RAG1* or *RAG2* gene to those identified in human patients are needed.

Here, we report the production and characterization of the *RAG1-86nt* Syrian hamster carrying a reading frameshift deletion disrupting the N-terminal non-core domain of RAG1 to recapitulate a subtype of the genetic mutations of Omenn syndrome. Western blotting experiments showed that, similar to RAG1 expression in humans, the hamster RAG1 is expressed in two isoforms, most likely by alternative usages of methionine (Met) as start codons of translation, i.e., a full length RAG1 from the first Met and a N-terminal truncated RAG1 by the use of the second Met site; the introduction of the 86nt deletion renders both of the isoforms undetectable by Western blotting. We further showed that, among other immunological defects, the development of T and B lymphocytes in the *RAG1-86nt* hamsters is severely impaired, as evidenced by the barely detectable CD3^+^ and CD4^+^ T cells and the dramatically reduced expression of B lymphocyte-specific markers in lymphatic organs. Furthermore, we challenged the *RAG1-86nt* hamsters with HAdV-C6 via intranasal infection and demonstrated that these hamsters can still mount anti-adenovirus humoral immunity, though with slower kinetics in clearing the infectious viruses.

## 2. Materials and Methods

### 2.1. Animals

Syrian hamsters used in gene targeting studies were produced by breeding animals purchased from Charles River Laboratories at the Laboratory Animal Research Center at Utah State University. The animals were housed in polycarbonate isolator cages and fed irradiated chow and water. For *RAG1-86nt* hamsters, drinking water was supplemented with 0.1 mg/mL enrofloxacin (Baytril, Bayer, Whippany, NJ, USA).

### 2.2. Ethics Statement

The experiments were conducted in strict accordance with guidelines of the AAALAC accredited Laboratory Animal Research Center at Utah State University and approved by the Institutional Animal Care and Use Committee of Utah State University (IACUC Protocol: 2763; approval period: 3 August 2017 through 2 August 2018). All surgery was performed under Ketamine/Xylazine anesthesia, and all efforts were made to minimize animal suffering.

### 2.3. Embryo Manipulation and Genotyping of Pups Produced from Injected Embryos

Embryo manipulation, embryo transfer, and post-surgery care were performed as described by us previously [12,13]. Briefly, female Syrian hamsters at 8 to 12 weeks of age were used both as embryo donors and recipients. Females were superovulated by intraperitoneal (ip) injection of 10–20 IU of PMSG (Sigma-Aldrich, St. Louis, MO, USA) according to their bodyweights at 9:00 AM on Day 1 of the estrous cycle. At 7:00 PM on Day 4 of the estrous cycle, females were mated with fertile males for zygote production or with vasectomized males for pseudo-pregnancy establishment. Zygotes were flushed from oviducts with HECM-9 medium approximately 19 h after mating. Zygotes were collected and transferred into 20 μL drops of HECM-9 covered by mineral oil (Sigma-Aldrich) in a culture dish and cultured at 37.5 °C under 10% CO_2_, 5% O_2_, and 85% N_2_ before use. Microinjection of hamster zygotes was carried out in a room with a small incandescent lamp where direct exposure of the embryos to light was avoided. A group of 15–20 zygotes was transferred to a 100 μL HECM-9 drop on the microinjection dish, and 1–2 pL of ribonucleoprotein (RNP; 100 ng/μL Cas9 protein and 50 ng/μL gRNA) solution was injected into the pronuclei of the zygotes. Injected embryos were washed twice with HECM-9 and cultured in HECM-9 covered by mineral oil for 0.5 h before embryo transfer. Embryos that survived injections were transferred to the oviducts (10–15 embryos per oviduct) of pseudo-pregnant females which were allowed to naturally deliver and raise their pups.

Genotyping analysis of pups produced from microinjected embryos was performed by PCR restriction fragment length polymorphism (PCR-RFLP) assay with genomic DNA isolated from ear biopsies collected from two-week-old pups. Genotyping PCR primers are: Forward1: 5′-AGACAAAGCAATTCACCAAG-′3 and Reverse1: 5′-ATCGGAGAATGCAGATTCTG-′3. Ex Taq Kit (Takara, Mountain View, CA, USA) was used following the manufacturer’s recommendations. PCR reactions were performed in a volume of 50 μL with the following programs: 95 °C for 3 min; 35 cycles: 98 °C for 10 s, 57 °C for 30 s, 72 °C for 40 s; 72 °C for 3 min; 4 °C ∞. The 662 bp PCR products of the WT allele were cleaved by TaqI (Thermo Fisher Scientific, Waltham, MA, USA) into 469 bp and 191 bp fragments, while the PCR products amplified from *RAG1*-targeted allele remained unchanged.

### 2.4. Western Blotting

Whole cell protein extracts were isolated from spleen and thymus from WT and *RAG1* targeted Syrian hamsters (*n* = 3) with NP40 buffer supplemented with Complete Mini Protease Inhibitor Cocktail Tablets (Roche, Indianapolis, IN, USA), sodium orthovanadate (NEB, Ipswich, MA, USA), PMSF (Sigma-Aldrich) and DTT (Sigma-Aldrich). Bradford Assay was used to estimate the protein concentration. 50 μg of protein supplemented with 5× LSB loading dye was separated in a 7.5% gel by SDS-PAGE and transferred to Turbo Midi PVDF by semi-dry blotting. After blocking for 1 h at room temperature with a blocking solution of 5% dry milk, the membrane was incubated with the first antibody diluted in blocking solution at 4 °C overnight. Incubation with the secondary, HRP-conjugated antibodies was done for 1 h at room temperature. For detection of the RAG1 protein, a 1:500 dilution of the RAG-1(D-5) antibody (sc-377127, Santa Cruz Biotechnology, Santa Cruz, CA, USA) was used as the first antibody and a 1:12,000 dilution of a goat anti-mouse IgG (HRP) was used as the secondary antibody (ab205719, Abcam, Cambridge, MA, USA). β-actin expression was demonstrated with a 1:12,000 dilution of beta actin (IAC-15) antibody (ab6276, Abcam) as the first antibody and a 1:12,000 dilution of the goat anti-mouse antibody (ab205719, Abcam) as the second antibody.

### 2.5. Histology and Immunohistochemistry

Spleen samples were fixed in 10% neutral buffered formalin for 24 h, after which the samples were transferred to 70% ethanol. All further processing of the samples was performed at the Histopathology & Tissue Shared Resource (HTSR) at Georgetown University (via Science Exchange). Immunohistochemical (IHC) staining for the hamster CD3ε protein was done using a 1:100 dilution of M-20 antibody (Santa Cruz Biotechnology).

### 2.6. Flow Cytometry

Single cell suspensions of spleen were incubated in Pharm Lyse buffer (Beckton Dickinson (BD), Franklin Lakes, NJ, USA) to lyse erythrocytes. The cells were stained with Live/Dead Fixable Aqua (Life Technologies, Grand Island, NY, USA) and fluorochrome-conjugated antibodies against CD4 and CD3ε (1:200 dilution of A700-conjugated anti-mouse CD4 (clone L3T4, E-Bioscience, San Diego, CA, USA) and 1:1000 dilution of APC-conjugated M-20, respectively). The samples were analyzed on a BD Biosciences LSR II. The data was acquired using FACSDiva software (v6.1.3, BD) on the LSR II, and analyzed using the FlowJo software (v9.5.1, BD).

### 2.7. Reverse Transcriptase Quantitative PCR (RT-qPCR)

Total RNA from spleen was extracted using the RNeasy mini kit (Qiagen, Valencia, CA, USA). All RNA samples were treated with RNase-free DNase followed by RNA cleanup to eliminate DNA contamination. The RNA yield was determined on a NanoDrop-2000 spectrophotometer (Thermo Fisher Scientific). For RT-qPCR, 2 μg of each RNA and 50 pM of oligo(dT) primer were used for the reverse transcription (RT) using High Capacity cDNA Reverse Transcription kit (ABI, Forster City, CA, USA). SYBR-green-based qPCR was used to specifically detect target gene mRNA. RT-qPCR primers for each of the genes analyzed are listed in Table 1. The data were analyzed using the ΔΔCt method. Housekeeping gene RPL18 was used as an endogenous control for normalization. The final value is displayed as the relative fold change between the WT and *RAG1-86nt* hamsters.

### 2.8. In Vivo Infection of HAdV-C6 in Wild Type and RAG1-86nt Syrian Hamsters

Hamsters were infected intranasally by instilling 3 × 10^10^ plaque forming units (PFU) per kg of HAdV-C6 into the nostrils of the animals in 100 μL PBS under isoflurane (Isothesia, Henry Schein Animal Health, Dublin, OH, USA). Control animals were administered PBS. Hamsters were observed and weighed daily; moribund animals were sacrificed as needed. At necropsy, lungs were collected and the infectious virus burden was determined using a 50% Tissue Culture Infectious Dose (TCID_50_) assay as described previously [14].

### 2.9. Determining the Anti-Ad6 Neutralizing Antibody (NAb) Titers in the Serum

The NAb titer of the samples was determined as described previously [15]. Briefly, complement in the serum samples were inactivated at 56 °C for 30 min. HAdV-C6 was incubated with dilutions of the serum samples, and then the virus-serum mix was added to A549 cells and incubated at 37 °C. After 10 days, the inhibition of cytopathic effect was measured by staining the cells with neutral red. The NAb titer was calculated as the reciprocal dilution causing 50% inhibition of viral cytopathic effect.

### 2.10. Statistical Analysis

Statistical analysis was performed using GraphPad Prism 7 (GraphPad Software, La Jolla, CA, USA). Comparison between groups was performed using the unpaired *t*-test with Welch’s correction. *p* ≤ 0.05 was considered significant.

## 3. Results

### 3.1. Genetic Targeting of the Non-Core Domain of Hamster RAG1 Protein

**Figure 1:**
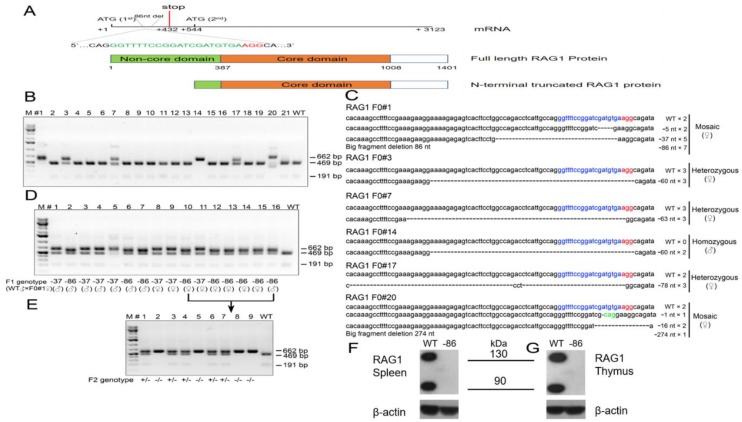
Generation of *RAG1*-targeted Syrian hamster with the CRISPR/Cas9 system. (**A**) Schematic of the Syrian hamster *RAG1* mRNA and the full length and N-terminal truncated isoforms of RAG1 proteins. In the mRNA diagram, the sgRNA target sequence is shown in green letters with the protospacer adjacent motif (PAM) shown in red. Also shown in the mRNA diagram is the positions of the 86-nt deletion, the first and second Met (ATG) sites, and the first premature stop codon caused by the 86-nt deletion. In the RAG1 protein diagram, the non-core domain and core domain are shown in green and apricot, respectively. (**B**) Genotyping results by the PCR-RFLP assay on 21 golden Syrian hamster pups produced by PN injections. (**C**) Indels introduced into the RAG1 locus of the 6 pups, #1, #3, #7, #14, #17 and #20. The frequencies of DNA subclones with each of the indels are indicated by numbers following the indel signs. (**D**) Genotyping results with the PCR-RFLP assay on the 16 F1 pups produced by breeding founder animal #1 with a wild type littermate. The sgRNA target sequence is shown in blue letters with the PAM shown in red; deletions are indicated by dashes, while altered nucleotides are marked green. (**E**) Genotyping results with the PCR-RFLP assays on the nine F2 pups produced by crossing the two littermates, #10 (female) and #16 (male). (**F**) The expression of RAG1 protein in the spleen of wild-type and homozygous 86-nucleotide deletion hamsters by Western Blot. (**G**) The expression of RAG1 protein in the thymus of wild-type and homozygous 86-nucleotide deletion hamsters by Western Blot.

To produce genetically engineered hamster models of SCID, the CRISPR/Cas9 system was employed to target exon 2 of the hamster *RAG1* gene encoding part of the N-terminal non-core domain of RAG1 (Figure 1A). As CRISPR/Cas9 can introduce either inframe or frameshift indels in a targeted gene, we reasoned that an inframe indel would disrupt the non-core domain but leave the core domain intact, mimicking a subset of the genetic defects in SCID, such as those observed in certain Omenn syndrome patients. On the other hand, a frameshift indel would introduce premature early stop codons interrupting the expression of the full-length RAG1 protein, modeling the genetic defects that fully abolish the function of RAG1 (a *RAG1*-null or KO genotype) as observed in more extreme forms of SCID; it is also possible that a frameshift indel in the non-core domain may still allow the alternative usage of other Mets (ATG) downstream of the indels as translation start codons to produce N-terminal truncated RAG1 with partial VDJ recombination activities, as observed in some Omenn syndrome patients [16]. With such intentions in mind, we conducted pronuclear (PN) injections of sgRNA/Cas9 ribonucleoprotein (RNP) specifically targeting exon 2 of hamster *RAG1* into hamster zygotes, followed by transferring the injected embryos to pseudopregnant female hamsters for the production of genetically engineered hamsters. From the 119 injected zygotes, 103 survived, and 87 were transferred to three pseudopregnant surrogate females (Table 2). All of the three surrogate mothers became pregnant and together gave births to 21 live pups, all of which survived to adulthood. Genotyping analysis with a PCR-RFLP assay identified that six of the 21 pups carry targeted mutations in the *RAG1* gene (Figure 1B). To reveal the identities of the indels in each of these six pups, we subcloned the PCR products amplified with the same PCR primers used in the PCR-RFLP assay into pGEM-T Easy vectors and subjected them to Sanger sequencing. As shown in Figure 1C, F0#1 (female) is a mosaic carrying alleles with 5-, 37- and 86-nucleotide (nt) deletions, as well as a wild type (WT) allele; animal F0#3 (female) carries a 60-nt deletion allele and a WT allele; animal F0#7 (female) carries a 63-nt deletion allele and a WT allele; animal F0#14 (male) carries a 60-nt deletion allele; animal F0#17 (female) carries a 78-nt deletion allele, a 3-nt change allele and a WT allele; animal F0#20 (female) is a mosaic carrying alleles with 1-, 16- and 274-nt deletions and a WT allele. All of these indels in each of the founder hamsters interrupt the coding sequence of the non-core domain of the hamster RAG1 protein, resulting in either amino acid deletions in this domain (the inframe indels) or the introduction of premature stop codons downstream of the indels to potentially generate a *RAG1*-null genotype. Because all of the indels identified in F0#1 (5-, 37- and 86-nt deletions) have resulted in reading frameshifts in the *RAG1* gene leading to multiple premature stop codons, to establish hamster colonies carrying indels that fully abolish the expression of full-length RAG1, we chose to breed F0#1 with a wild type male. Each of the 16 F1 pups (all from the same litter) from this breeding was identified as heterozygous with an indel by the PCR-RFLP assay, demonstrating germ line competence of the indels. Sanger sequencing of the PCR products from each of the F1 pups after subcloning into the pGEM-T Easy vector demonstrated that both of the 37- and 86-nt deletion alleles were transmitted to the germline (Figure 1D). With a sufficient number of F1 pups carrying either the 37-nt deletion or the 86-nt deletion, we did not try to breed F0#1 again to produce more pups to demonstrate whether the 5-nt or the WT allele have also transmitted to the germline. To establish a hamster colony homozygous for the 86-nt deletion, we conducted a sister-brother breeding by using the F1 hamsters carrying the 86-nt deletion. Heterozygous *RAG1*-*86nt* hamsters showed normal reproduction with average litter sizes ranging from 6 to 12. The 86-nt deletion was inherited in the expected Mendelian ratio. Figure 1E shows the PCR-RFLP assay results from a litter of F2 pups. By using some of the *RAG1-86nt* homozygous F2 hamsters, we have successfully established a *RAG1*-*86nt* hamster breeding colony. Homozygous *RAG1-nt86* hamsters also show normal reproduction with similar litter size as with WT and heterozygous animals. Hamsters from other F0 founders that carry inframe indels, such as F0#3 carrying a 60-nt deletion and F0#7 carrying a 63-nt deletion allele, will be characterized and reported elsewhere.

### 3.2. The 86-nt Deletion Abolishes the Expression of Full-Length RAG1 Protein

As shown in Figure 1A, the 86-nt deletion, from position 413 to 499, in the *RAG1* gene caused a reading frameshift and generated multiple premature stop codons with the first one located at position 432 of the *RAG1* mRNA, thereby disrupting the expression of the full length RAG1 protein and possibly leading to a *RAG1* KO phenotype. However, there are multiple methionins downstream of the 86-nt deletion which, if used as translation start codons as in human *RAG1* [16], could generate truncated RAG1 proteins which lack the N-terminal domain (Figure 1A). In light of this, we chose to use an anti-RAG1 antibody recognizing the C-terminus of the RAG1 protein as the primary antibody for the Western blotting assay to detect RAG1 expression. We reasoned that if a truncated form of RAG1 protein lacking the N-terminal non-core domain is produced, it still should be detectable in the *RAG1-86nt* hamsters. As speculated, Western blotting experiments using thymus and spleen lysates prepared from wild type pups detected two bands, one being about 135 kD and the other 95 kD, while neither of these two bands were detectable in the spleen and thymus cell lysates derived from the littermates homozygous with 86-nt deletion (Figure 1F). We concluded that, similar to what has been observed in humans, the hamster RAG1 protein is indeed expressed in two isoforms by the alternative usage of methionins, a full length 135 kD from using the first Met and a truncated 95 kD isoform from using Met-182 as the translation start codon, respectively. Because the 86-nt deletion introduced multiple premature stop codons in *RAG1*, the expression of the full length RAG1 must have been fully abolished in the *RAG1-86nt* hamsters. The disappearance of the 95 kD band in the Western blot indicates that the 86-nt deletion also affects the expression of the truncated form of RAG1, possibly due to less stable mRNA carrying the 86nt deletion or other mechanisms (see Discussion below).

### 3.3. Off-Target Effect Analysis

**Figure 2:**
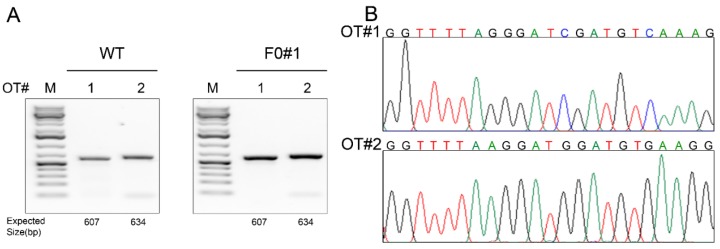
Off-target effect analysis. (**A**) PCR products flanking each predicted off-target (OT) site (#1–2) in wildtype (WT) and a founder (F0#1) hamster. (**B**) Sanger sequencing results of the PCR products showing that no off-target indel was introduced to either of these two sites.

Even though the gRNA used in this study scores highly in target-specificity accordingly to the sgRNA design tool at Benchling, we nevertheless conducted off-targeting analysis. We used the sgRNA target sequence as the query to blast the Syrian hamster genome assembly (https://www.ncbi.nlm.nih.gov/genome/11998) to identify any sequences in the hamster genome that share a homology with the target sequence with up to two nucleotide mismatches in the seed sequence [17]. With this homology criterion, we identified only two potential off-site targets (OT1 and OT2) (Table 3). We designed PCR primers for these two genomic loci and conducted PCR-RFLP assays by using the genomic DNA isolated from each of the F0 founders and a WT hamster (as a control) and subjected the PCR products to Sanger sequencing. As shown in Figure 2A,B for founder F0#1, there was no off-target event detected in any of the F0 founders.

### 3.4. RAG1-86nt Hamsters Are Atrophic in Lymphoid Organs

**Figure 3:**
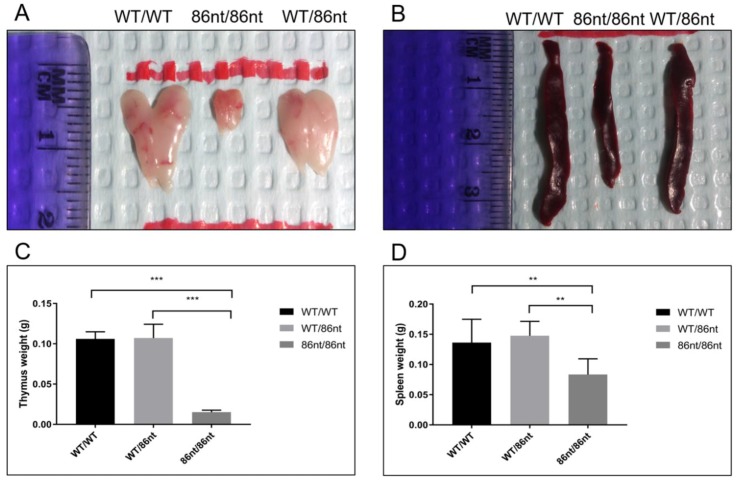
Analysis of organ sizes and weights. (**A**) and (**B**) thymus and spleen of *RAG1-86nt* hamsters were both evidently smaller than that of age-matched *RAG1-86nt*-heterozygous and WT hamsters. (**C**) Comparison of thymus weights, *** *p* < 0.001. *n* = 4. (**D**) Comparison of spleen weights, ** *p* < 0.01. *n* = 4.

**Figure 4:**
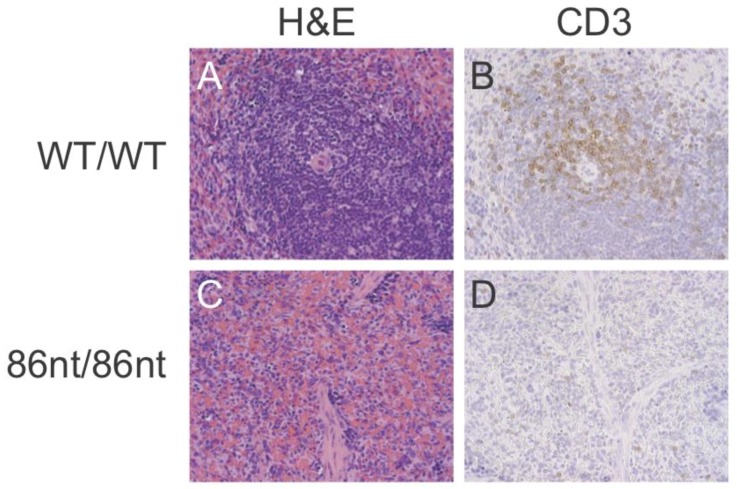
Histological analysis of spleen tissues. (**A**,**C**) hematoxylin-eosin staining. (**B**,**D**) immunohistochemical staining for CD3ε. *RAG1-86nt* hamsters had atrophic spleens and only a very small number of CD3^+^ cells.

To investigate if the abolishment of the full length RAG1 protein expression affects lymphatic tissue development in the hamster, we examined the thymuses and spleens in age-matched (at 6-week of age) *RAG1-86nt*-homozygous, -heterozygous, and WT hamsters. As shown in Figure 3A,B, gross necropsy showed that the thymus and spleen of the *RAG1-86nt*-homozygous hamsters are significantly reduced in sizes compared to *RAG1-86nt*-heterozygous and WT controls. The spleens of *RAG1-86nt*-homozygous hamsters were much thinner, shorter and more loosely packed than those of age-matched heterozygous and WT hamsters. The mean thymus weight was significantly reduced from 0.106 g (SD ± 0.09) in WT and 0.107 g (SD ± 0.02) in *RAG1-86nt*-heterozygous hamsters to 0.015 g (SD ± 0.02) in *RAG1-86nt*-homozygous hamsters (Figure 3C). The mean spleen weight was significantly reduced from 0.136 g (SD ± 0.04) in WT and 0.148 g (SD ± 0.02) in *RAG1-86nt-*heterozygous hamsters to 0.083 g (SD ± 0.03) in *RAG1-86nt-*homozygous (Figure 3D). Histologic staining with hematoxylin-eosin (H&E) confirmed that the spleen of *RAG1-86nt*-homozygous hamsters contained less white pulp than that of their WT counterparts, and was almost completely devoid of lymphoid follicles (Figure 4A,C).

### 3.5. Naïve RAG1-86nt Hamsters Have Severely Reduced Size of T and B Lymphocyte Compartments

**Figure 5:**
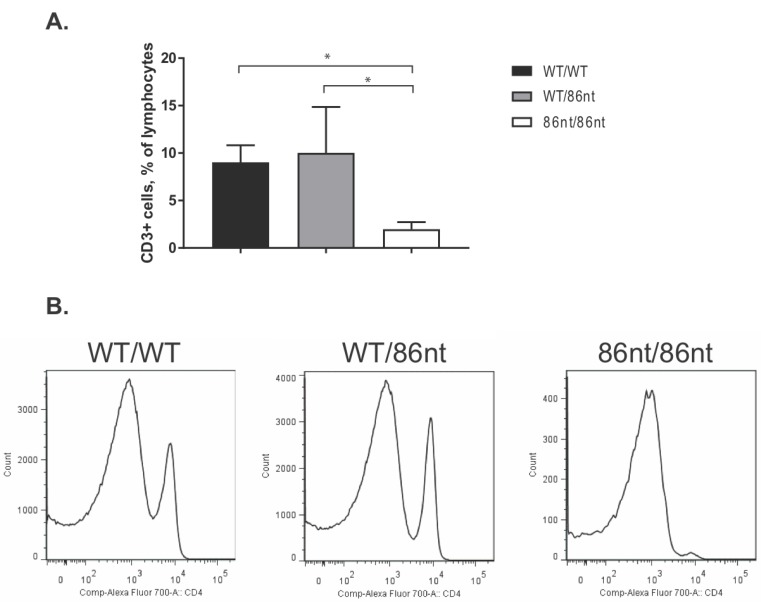
Flow cytometry analysis of CD3^+^ and CD4^+^ cells in the spleen. (**A**) Significantly less CD3^+^ cells were detected in the spleen of *RAG1-86nt* hamsters (*n* = 3 for all groups). (**B**) Staining for CD3^+^ and CD4^+^ cells (representative images are shown). * *p* < 0.05.

**Figure 6:**
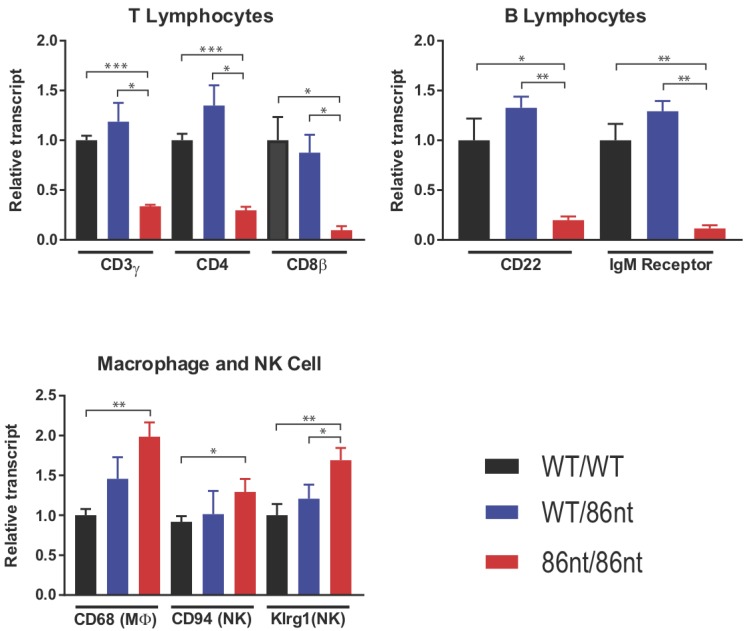
Quantitative RT-PCR analysis of lymphocyte-specific genes in the spleen. Lower levels of mRNAs for T and B lymphocyte-specific genes were detected, while the relative amount of macrophage and NK cell-specific transcripts is higher in the spleen of these animals. The amount of the indicated transcripts in spleen homogenates was determined by quantitative RT-PCR using the ∆∆Ct method. *n* = 3 for all groups. * *p* < 0.05, ** *p* < 0.01, *** *p* < 0.001.

Immunohistochemical (IHC) staining for CD3ε in the spleen demonstrated that CD3 positive cells were barely detectable in *RAG1-86nt*-homozygous hamsters and that the T cells were randomly distributed in the spleen (Figure 4D). This finding was further corroborated by flow cytometry analysis, showing that *RAG1-86nt*-homozygous hamsters had practically no CD3^+^, CD4^+^ cells (helper T lymphocytes) in the spleen (Figure 5). Furthermore, splenocytes isolated from *RAG1-86nt*-homozygous hamsters expressed considerably less T lymphocyte-specific (CD3γ and CD4) and B lymphocyte-specific (CD22 and FCMR (IgM receptor)) transcripts than WT ones (Figure 6). Interestingly, the relative expression of macrophage-specific (CD68) and natural killer (NK) cell-specific (CD94 and Klrg1) markers was increased in the spleen of *RAG1-86nt*-homozygous hamsters compared to that of WT animals (Figure 6). Based on these findings, we concluded that *RAG1-86nt*-homozygous hamsters have atrophic lymphoid organs.

### 3.6. RAG1-86nt Hamsters Develop a Partial Adaptive Immune Response after Intranasal Infection with HAdV-C6

**Figure 7:**
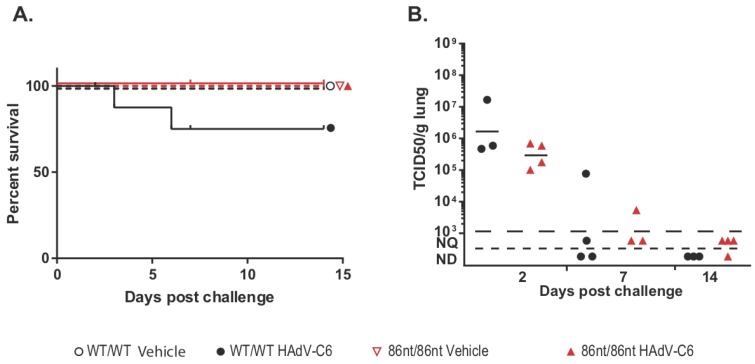
Survival and lung virus loads post intranasal infection by HAdV-C6. (**A**) Comparison of survivals among wild type (WT), *RAG1-86nt* heterozygous and *RAG1-86nt* homozygous hamsters following intranasal HAdV-C6 infection. (**B**) TCID50 of viruses recovered from lungs. The virus burden in the lungs of *RAG1-86nt* hamsters was marginally lower than that in the lungs of WT animals. For this and subsequent similar figures, symbols represent data from individual animals, and the horizontal bar indicates the geometric mean. NQ: not quantifiable; ND: not detectable.

**Figure 8:**
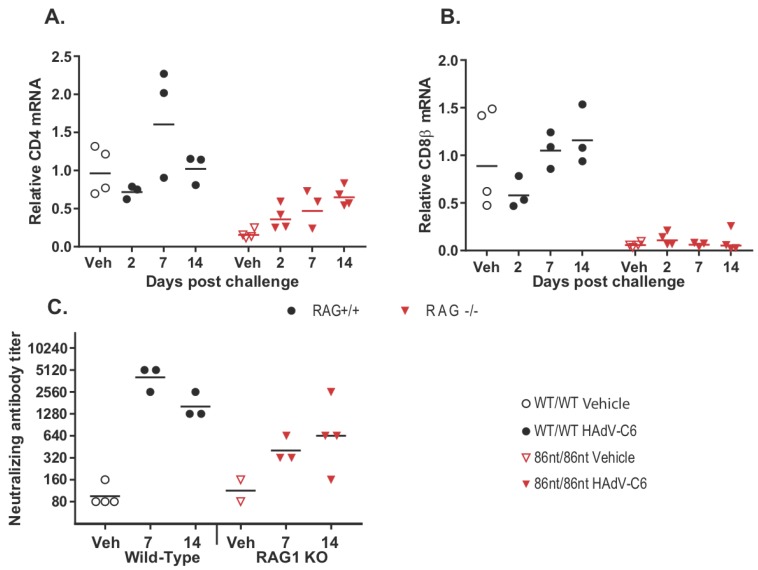
Adaptive immune response to intranasal HAdV-C6 infection. (**A**) infiltration of CD4^+^ and (**B**) CD8^+^ cells into the infected lung tissues; (**C**) development of neutralizing antibodies against HAdV-C6 infection. *RAG1-86nt* hamsters developed an imperfect adaptive immune response to HAdV-C6. Veh: vehicle-treated.

While human adenoviruses usually are not a great concern in healthy human populations, they can lead to severe clinical manifestations in immunocompromised patients [18]. Therefore, we were interested in investigating whether the *RAG1-86nt* hamsters would be more susceptible to human adenovirus infection. To this end, we infected *RAG1-86nt* and WT hamsters intranasally with HAdV-C6 and observed the animals throughout a 15-day period. Interestingly, opposite to our expectations, the *RAG1-86nt* hamsters survived better than WT hamsters following HAdV-C6 infection (Figure 7A); the infectious virus recovery in the lungs of *RAG1-86nt* hamsters was marginally lower than in WT hamsters (Figure 7B). We further investigated the functional mechanisms for this and found that the less severe disease outcome in the *RAG1-86nt* hamsters was associated with relatively lower levels of infiltration of CD4 and CD8 cells in the infected lung tissues in *RAG1-86nt* hamsters than in WT hamsters (Figure 8A,B). Surprisingly, *RAG1-86nt* hamsters developed neutralizing antibodies against HAdV-C6 (Figure 8C), though at lower levels than those in WT hamsters (Figure 8C), indicating that the low level of humoral response may be sufficient to clear the infectious virus.

## 4. Discussion

Mutations in the *RAG* genes, depending on the types or the locations in the *RAG* genes, cause SCID with a wide spectrum of disease manifestations. Both of the RAG1 and RAG2 proteins have functional domain structures that can be divided into core and non-core domains, with the core domains being the minimal region required for catalyzing V(D)J recombination and the non-core domains exerting a variety of regulatory functions, including nuclear import, interaction with other cellular proteins, and protein turnover [19]. Genetic mutations in the core domain tend to lead to more severe SCID phenotypes, while mutations in the non-core domain may lead to relatively mild disease presentations as seen in Omenn syndrome patients.

Mouse *RAG1* KO models have provided significant knowledge advancement in understanding the function of RAG1 proteins in B and T cell development and the consequences of a loss of the RAG1 function in the immune system. *RAG1* KO models in several other species have also been produced with the understanding that, due to the difference in the immune systems between human and animal species, a single animal model may not be able to recapitulate the entire aspect of immune dysfunction caused by *RAG1* mutations in human. It is most likely that each of these genetic animal models will offer some unique insights into the biology of RAG1 and will collectively contribute to the understanding of SCID caused by the loss of function of RAG1.

The Syrian hamster is an animal model of choice for several human diseases, especially for infectious diseases [6,7], a fact that bears some relevance to infections in SCID patients. It has been demonstrated by us and others that human cytokines, such as granulocyte–macrophage colony-stimulating factor (GM-CSF) and interleukin-12 (IL-12), are functional in the hamster but not in the mouse [20,21]. Other advantages in using the hamster to model certain infectious diseases is that the disease manifestations in the hamster are highly representative of what is observed in humans [7].

We recently succeeded in establishing genetic engineering technologies in the hamster and have produced several genetically engineered hamster models [12]. These hamster models have been successfully used to model several human diseases for which the other rodent models are not adequate [15,22,23]. In this study, we report the preliminary characterization of a genetically engineered hamster strain carrying an 86-nt frameshift mutation in the *RAG1* gene encoding part of the N-terminal non-core domain of RAG1. We demonstrated that *RAG1-86nt* hamsters have splenic and thymic atrophy, with significantly impaired development of both B and T lymphocytes. However, to our surprise, the *RAG1-86nt* hamsters are capable of mounting humoral responses against HAdV-C6. These results suggest alternative Met site(s) may be used in the translation of hamster RAG1 and that the 86-nt deletion mutation, while should abolish the expression of the full length RAG1 by introducing multiple premature stop codons in the *RAG1* gene, may only partially reduce the expression of the N-terminal truncated isoform (which contains the entire the core domain). What is worth noting is that our Western blotting experiments demonstrated that, while the two RAG1 isoforms are readily detectable in WT hamsters, none of them was detectable in the *RAG1-86nt* hamsters. We concluded that the 86-nt deletion also affects the expression of the 95 kD truncated isoform of RAG1. Several possible mechanisms could be responsible for the undetectable expression of the truncated RAG1 protein. It was shown in humans that a truncated RAG1 expressed from a nonstandard ATG is unstable when expressed alone without the presence of the full length RAG1 [16]; therefore, it is possible that the expression of the truncated RAG1 isoform in the 86-nt deletion hamsters is not stable enough to be detected by Western blotting. Alternatively, a lower level expression of the 95 kD isoform could also be the result from less stable *RAG1* mRNA by the 86-nt deletion. Nevertheless, leaky expression of the truncated isoform which contains the entire core domain of RAG1 would be capable of catalyzing V(D)J recombination, even though maybe much less efficiently. Furthermore, other possible mechanisms of development of anti-adenovirus humoural immunity may be present in *RAG1-86nt* hamsters, such as production of oligoclonal B cell clones; the mutant *RAG1* in this non-core region may exert different effect on V(D)J recombination, with inefficient cleavage activity, like other mutant RAG1 mutant reported previously [24]. These warrant further investigations into characterization of this unique model that replicates a specific subset of *RAG1* mutations. 

Another interesting finding from our current study is that infectious virus recovery in the lungs of *RAG1-86nt* hamsters was marginally lower than in WT hamsters (Figure 7B) and that the *RAG1-86nt* hamsters survived slightly better than WT hamsters upon HAdV-C6 infection (Figure 7A). Our RT-qPCR showed that the *RAG1-86nt* hamsters expressed lower levels of CD4 and CD8 (Figure 8A,B; vehicle animals) and that the levels of these genes either did not increase at all (CD8) or increased to a lower extent than in WT hamsters (CD4) over the time course of HAdV-C6 infection (Figure 8A,B), indicating a decreased infiltration of CD4^+^ and CD8^+^ cells. While CD8β expression is restricted to cytotoxic T lymphocytes, CD4 is expressed by helper T lymphocytes and monocytes at some stages of development, thus the infiltrating CD4^+^ cells may represent either population. The increased infiltration of immune cells in the lungs of WT hamsters compared to the *RAG1-86nt* ones may explain the worse survival of WT hamsters observed in Figure 7A. We previously showed that immune-mediated pathogenesis is an important factor in lung pathology for this model [14,25]; thus, the increased infiltration with T and B lymphocytes in the WT hamsters may exacerbate the pathology of adenovirus infection. 

In summary, the *RAG1-86nt* hamster generated in this study could be used potentially as a model to study human immunodeficiency caused by mutations in the coding sequences for the non-core domain of RAG1, such as those identified in Omenn syndrome patients. Two murine models of Omenn Syndrome were reported previously and both of them recapitulated a subset of Omenn Syndrome, showing oligoclonal T cells and autoimmune-like manifestations [26,27]. The mouse model presented by Khiong et al. is a *Rag2* knockin mouse model carrying a Rag2R229Q mutation in the core region of, whereas Marrella’s model is a spontaneous mutation in the core region of Rag1 in C57BL/10 mice. In the *RAG1-86nt* hamster reported herein, we genetically disrupted the N-term non-core domain of RAG1 of the Syrian hamster to represent another subset of genetic causes for Omenn Syndrome. Compared to what observed in the mouse models, the *RAG1-86nt* hamster model does not show significant autoimmune-like manifestations. It is possible that the different pathogenesis presentations observed in the mouse models and our hamster model may reflect the different genetic mutations introduced into these models. Considering the fact that detailed pathogenesis caused by mutations in the N-terminal non-core domain of RAG1 gene (occurred in 19% Omenn Syndrome patients) has not yet been well characterized due to the lack of a suitable model, the *RAG1-86nt* hamster developed in this study may provide such a model. This novel hamster model, along with the *STAT2* KO model that we developed [12], may be useful for dissecting the contributions of adaptive immunity and innate immunity to the control of adenovirus infections and possibly for other pathogens in humans. Furthermore, this model also may be useful for the development of new gene therapies for treating patients carrying this specific subset of *RAG1* mutations.

## Figures and Tables

**Table 1:** PCR primers for PCR-RFLP and RT-qPCR assays.

Category	Name	Sequence (5′-3′)	Note
For designated target	GH RAG1-F	AGACAAAGCAATTCACCAAG	For sgRNA/Cas9-RAG1
	GH RAG1-R	ATCGGAGAATGCAGATTCTG	For sgRNA/Cas9-RAG1
For potential off-site targets	OT1F	GGTTGACCTCTGGCCTAGAC	
	OT1R	CCTGTGCTTTGTTGGTTGTG	
	OT2F	CCTATCCGCATTGTCCCATC	
	OT2R	ATGGGGCTAATCTGGCGCAG	
For RT-qPCR	CD4-F	CATCGTAACCCAGAACCAGAAA	
	CD4-R	CCCTCGTATAGACTGTGGTAGAT	
	CD8β-F	CCAGAATCCAGAACCACTCACA	
	CD8β-R	CGGTCCAAGAAGGGTACAAGAA	
	CD3ʏ-F	GAGCACAATCAGTCCCTACAA	
	CD3ʏ-R	CTTTGCTCCTTGACACCAATAAG	
	CD22-F	CTGCAAGAGGTCTTCCTGTATC	
	CD22-R	GAAGACTCGATCCTGGTGTTATT	
	IgM Receptor-F	ACCTCAGTTCAGCAAGCAATC	
	IgM Receptor-R	CCTTGGTTCTCTCTCCCATACT	
	CD94-F	CTCATCTCTAGTGTGCTTGGTG	
	CD94-R	ATGGGACATGTTCTTTCAGGAG	
	Klrg1-F	GAGGAATGGTAGCCACTGTTT	
	Klrg1-R	TGTAAGGAGATGTGAGCCTTTG	
	CD68-F	CCTGTCTCTCTCGTTTCCTTATG	
	CD68-R	GTGGGAAGGACACGTTGTATT	
	RPL18-F	GTTTATGAGTCGCACTAACCG	
	RPL18-R	TGTTCTCTCGGCCAGGAA	

**Table 2:** Production of RAG1 targeted golden Syrian hamsters by pronuclear (PN) injection.

No. Inject Zygotes	No. Survived	No. Embryos (Pups)	No. Litters (Recipients)	No. Positive Pups (%)
119	103	87 (21)	3 (3)	6 (28.6)

**Table 3:** Top two potential off-targets by sgRNA/Cas9-RAG1.

	Off-Targets Sequence (5′-3′)	PAM	Off-Target Score	Sequence ID	Location of Sites
OT1	GGTTTTAGGGATCGATGTCA	AAG	0.7	NW_004801621.1	2321825 to 2321847
OT2	GGTTTTAAGGATGGATGTGA	AGG	0.7	NW_004801607.1	1888496 to 18885018

Nucleotide mismatches between the target sequence and the potential off-target sequences are in red letters. Our scoring calculation follows Feng Zhang Lab’s method (http://crispr.mit.edu/). Score is from 0–100. We also include potential off-target matches with NAG and NGG as PAMs.
